# Boron-Doping Proximity Effects on Dislocation Generation during Non-Planar MPCVD Homoepitaxial Diamond Growth

**DOI:** 10.3390/nano8070480

**Published:** 2018-06-29

**Authors:** Fernando Lloret, David Eon, Etienne Bustarret, Alexandre Fiori, Daniel Araujo

**Affiliations:** 1 Department of material science and ME and IQ, University of Cádiz, Puerto Real, 11510 Cádiz, Spain; daniel.araujo@uca.es; 2 Institute for Material Research, Hasselt University, 3590 Diepenbeek, Belgium; 3 IMOMEC, IMEC vzw, 3590 Diepenbeek, Belgium; 4 Univ. Grenoble Alpes, CNRS, Intitut Néel, F-38000 Grenoble, France; david.eon@neel.cnrs.fr (D.E.); etienne.bustarret@neel.cnrs.fr (E.B.); 5 National Institute for Materials Science (NIMS), International Center for Young Scientist (ICYS), 1-1 Namiki, Tsukuba, Ibaraki 305-0044, Japan; fiori.alexandre@nims.go.jp

**Keywords:** diamond, MPCVD, TEM, diamond growth, dislocations, boron-doped diamond, lateral diamond growth, selective diamond growth

## Abstract

Epitaxial lateral growth will be required if complex diamond-based device architecture, such as, for example, Metal-oxide-semiconductor Field-effect transistors (MOSFETs) or epitaxial lateral overgrowth (ELO) substrates, need to be developed for high-power applications. To this end, undoped and doped non-planar homoepitaxial diamond were overgrown on (001)-oriented diamond-patterned substrates. Defects induced by both the heavy boron doping and three-dimensional (3D) growth were studied by transmission electron microscopy (TEM). At high methane and boron concentrations, threading dislocations with Burgers vectors **b** = 1/6 〈211〉, **b** = 1/2 〈110〉, or both were observed. Their generation mechanisms were established, revealing boron proximity effects as precursors of dislocations generated in boron-doped samples and providing clues as to the different Burgers vectors. The concentration ranges of boron and methane resulting in good crystalline quality depended on the plane of growth. The microwave plasma-enhanced chemical vapour deposition (MPCVD) growth conditions and the maximum boron concentration versus plane orientation yielding a dislocation-free diamond epitaxial layer were determined.

## 1. Introduction

Wide-band-gap materials (WBGMs) are expected to replace silicon for power electronic applications. Properties such as their much higher breakdown field, thermal conductivity, or energy band-gap imply possibly huge benefits in commercial power devices [1]. SiC power devices are already established, with a $11.5 billion market in 2014, and III–V semiconductors such as GaN are reaching a similar level of prevalence [2]. Synthetic diamond has lagged behind despite having the best intrinsic properties among the WBGMs [3,4]. Over recent years, the progress of microwave plasma-enhanced chemical vapour deposition (MPCVD) diamond for power electronic applications has resulted in the fabrication of a Schottky diode with extremely good rectification behavior and a high current density, about 10^3^ A·cm^−2^ at 6 V; a very low reverse current density, 10^–9^ A·cm^–2^, up to the maximum voltage |*V*_max_| = 1000 V; and the highest Baliga’s power figure of merit (FOM) reported up to now (244 MW·cm^–2^) [5]. However, crystalline quality or electrical contacts are still issues that need to be improved in order to attain competitive diamond commercial devices. Moreover, the difficulties in obtaining high-quality diamond substrates and the reduced dimensions of those commercially available have blurred their industrial prospects.

In Si, SiC, and III–V materials, epitaxial lateral overgrowth (ELO) has shown very good results in terms of defect reduction over large areas as well as great advantages for electronic devices, such as power efficiency, performance enhancement, product miniaturisation, cost reduction, and modular design for improved time to market [6,7,8,9,10]. Such benefits are also expected from its implementation in diamond [11,12,13]. However, the lateral growth of diamond usually leads to a high density of defects. The conditions to reach selective boron-doped diamond lateral growth were recently obtained for {001}-oriented patterned substrates. Such growth conditions, that is, the use of low methane concentrations [14], resulted in a low growth rate (<12 nm·min^–1^) that justified further optimisation of the process. In fact, homoepitaxial boron-doped layers overgrown on both (001)- and (111)-oriented substrates usually contain lattice-related defects. Alegre et al. showed a direct relationship between the boron doping level and the dislocation density that depended on the plane of growth and on the methane concentration [15]. Whatever their origin, these defects have an undesirable impact on the resulting diamond-based devices and should be avoided. The optimisation of both growth rates and boron doping levels is thus one of the required technological steps toward commercial electronic devices based on three-dimensional (3D) diamond architectures.

## 2. Materials and Methods

**Figure 1:**
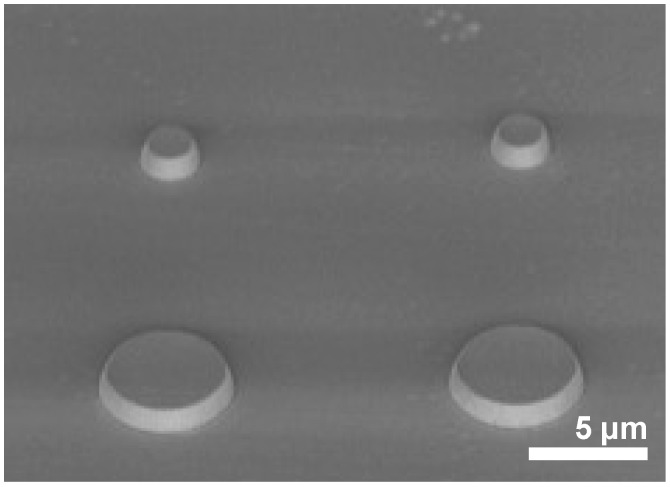
Scanning electron microscopy (SEM) micrograph of one of the substrates after inductively coupled plasma reactive ion etching (ICP-RIE) process.

The effect of the growth orientation and doping level on the dislocation generation was studied by transmission electron microscopy (TEM), with the aim to determine the best conditions for diamond selective growth. Four (100)-oriented high-pressure, high-temperature (HPHT) diamond substrates were patterned into 1 µm high mesa-shaped disks (see Figure 1) by inductively coupled plasma reactive ion etching (ICP-RIE), in order to make different growth orientations available in the same sample under exactly the same growth conditions. Growth was performed by MPCVD in a home-made NIRIM-type reactor [16]. Sample 1 was a multi-layered sample formed by a stack of 13 doped/undoped bilayers. Doped layers were grown with 0.25% CH_4_/H_2_ (molar) and 10,700 ppm B_2_H_6_/CH_4_ over 2 min. Undoped layers were grown for 60 min using 0.1% of methane in hydrogen. The pressure and plasma power were kept at 4.4 kPa and 300 W, respectively. Sample 2 was a boron-doped sample grown at 4% CH_4_/H_2_ and 1200 ppm B_2_H_6_/CH_4_ for 10 min. Sample 3 was also a doped-layer sample in which the methane concentration was reduced to 0.5%. Diborane in hydrogen (6000 ppm) was used for doping, and the growth time was 10 min. Finally, sample 4 was a multi-layered sample formed by a stack of 11 doped/undoped bilayers. Doped layers were grown with 0.5% CH_4_/H_2_ and 14,000 ppm B_2_H_6_/CH_4_ over 11 min, and undoped layers were grown over 12 min using 0.75% of methane in hydrogen and a 0.32% O_2_/H_2_ ratio.

The TEM study was performed on electron-transparent lamellas extracted from these samples by a focused ion beam (FIB) in a dual-beam FEI Quanta 200 3D microscope. The TEM studies were carried out in a field-emission JEOL 2010F and in a thermo-ionic filament-emission Philips CM200, both working at a 200 kV accelerating voltage.

## 3. Results

**Figure 2:**
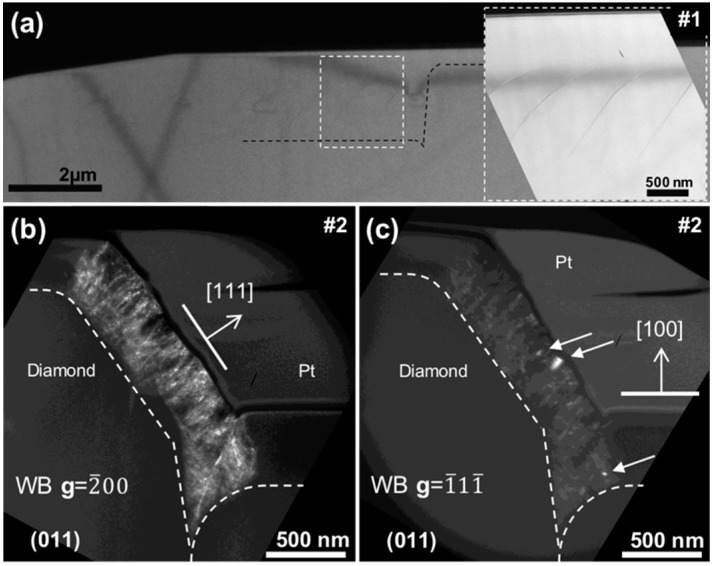
(**a**) Bright-field cross-section micrograph of sample 1 with the lamella oriented along the {011} pole. A black dashed line marks the initial shape of the etched cylinder. The region framed by a white dashed square is enlarged in the right-hand inset. (**b**) Weak-beam micrograph of sample 2 with the lamella oriented along the {011} pole, recorded under two beam conditions using the $\left[\bar{2}00\right]$ reflection. Lateral growth orientation is marked by a white arrow. (**c**) Weak-beam micrograph of sample 2 with the lamella oriented along the {011} pole, recorded under two beam conditions using the $\left[\bar{1}1\bar{1}\right]$ reflection. Three white arrows mark dislocations invisible in the $\left[\bar{2}00\right]$ reflection. White dashed lines mark the initial shape of the etched truncated cone (“disk”) in both micrographs. The vertical (and defect-free) [001]-oriented growth is also marked by a white arrow.

Figure 2a shows a bright-field (BF) TEM micrograph of sample 1 oriented along the [011] pole. A black dashed line marks the initial shape of the substrate, and doped layers are noted by smooth darker contrasts. The doped layers, with a boron content estimation of ~10^19^ atm·cm^–3^, are better seen in the magnified region of the inset. The sample was free of dislocation, and the promotion of the lateral growth is also clear, with a much larger thickness along the lateral side (a few micrometers) than along the vertical side (barely 100 nm). This was the result of a 12 nm/min growth rate along the <111> direction and of only 1 nm/min along <100>.

Figure 2b,c shows two weak-beam (WB) TEM micrographs of sample 2 oriented along the (011) pole, recorded under two beam conditions using the $\left[\bar{2}00\right]$ and $\left[\bar{1}1\bar{1}\right]$ reflections, respectively. White dashed lines mark the initial shape of the patterned substrate in both micrographs. This sample consisted of boron-doped layer overgrowth with 4% CH_4_/H_2_, and a boron content was estimated at 10^21^ atm·cm^–3^. These growth conditions resulted in a lateral growth rate of 38 nm/min, 3 times faster than for sample 1, and a [001]-oriented growth rate of 35 nm/min. A high density of dislocations was observed on the region grown laterally, that is, along the <111> direction. In contrast, the rest of the cross-sectional view, corresponding to growth along the <100> direction, appeared free of defects. Because the growth rates were very similar along both orientations, this situation established a different behaviour in the dislocation generation depending on the plane of growth. Burgers vectors of the threading dislocations were identified using the invisibility criterion [17], resulting in the majority of dislocations with a vector $b=\frac{1}{6}\langle 211\rangle$ almost homogeneously distributed over the layer and only a few others, marked with arrows, with Burgers vector $b=\frac{1}{2}\langle 011\rangle$. The density of dislocations, defined as the sum of dislocation lengths per volume [17], was calculated for each growth orientation of this sample, resulting in 4 × 10^10^ cm^–2^ in the <111> growth sector and none for the <100> sector.

**Figure 3:**
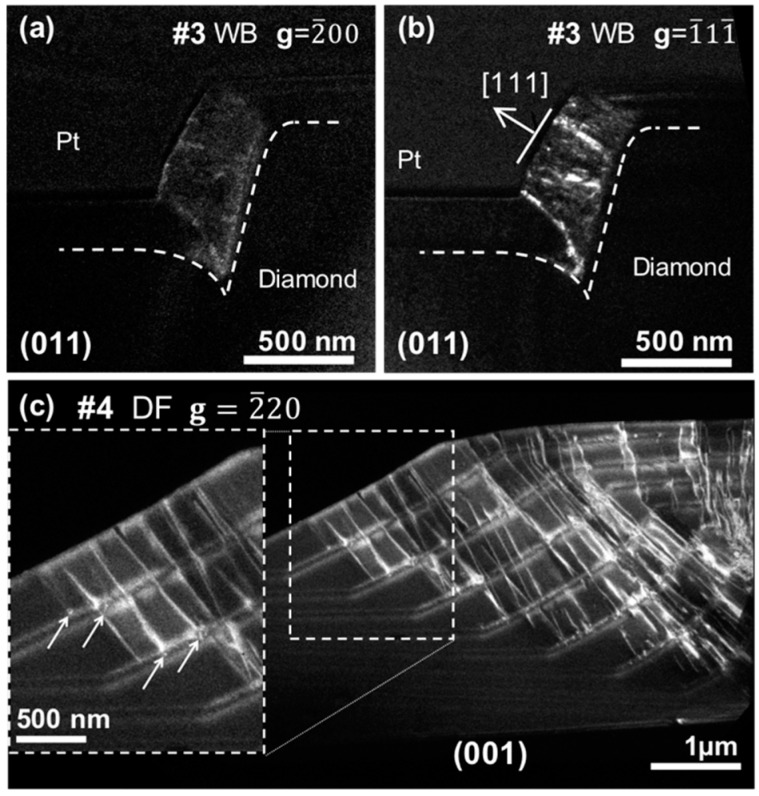
(**a**) Weak-beam micrograph of sample 3 with the lamella oriented along the {011} pole, recorded under two beam conditions using the $\left[\bar{2}00\right]$ reflection. (**b**) Weak-beam micrograph of sample 3 with the lamella oriented along the {011} pole, recorded under two beam conditions using the $\left[\bar{1}1\bar{1}\right]$ reflection. Lateral growth orientation is marked by a white arrow. White dashed lines mark the initial shape of the etched cylinder in both micrographs. (**c**) Dark-field micrograph of sample 4 with the lamella oriented along the {001} pole, recorded under two beam conditions using the $\left[\bar{2}20\right]$ reflection. A high density of defects is observed arising from the doped layer. This is clearer in the inset displaying an enlarged image of the white dashed–framed region. White arrows in the inset marks some of the dislocations generated in the doped layers.

Figure 3a,b shows WB micrographs of sample 3 oriented in the (011) pole, recorded using the $\left[\bar{2}00\right]$ and $\left[\bar{1}1\bar{1}\right]$ reflections, respectively. This sample was grown with a similar boron content, estimated at 3 × 10^21^ atm·cm^–3^, but with a lower methane/hydrogen ratio than for sample 2. The use of a lower methane concentration implied a higher growth selectivity, because, for this sample, the growth rates were 42 and 23 nm/min along the <111> and <100> directions, respectively. However, also in this case, micrographs showed the laterally grown region to contain many defects. In contrast, 001-oriented growth appeared free of dislocations. The dislocation distribution along the lateral growth layer also seemed different to that of sample 2. It was clearly inhomogenously distributed, and only dislocations with $b=\frac{1}{2}\langle 011\rangle$ Burgers vectors were obtained. The density of dislocations estimated in this sample was zero again for the <100> orientation and was relatively high, 25 × 10^10^ cm^–2^, in the <111> growth sector.

Figure 3c shows a dark-field (DF) micrograph of sample 4 in the (001) pole, recorded under two beam conditions using the $\left[\bar{2}20\right]$ reflection. This sample was multi-layered and grown with a boron content similar to those of samples 2 and 3, estimated at 2.3 × 10^21^ atm·cm^–3^. The methane/hydrogen molar ratio remained at 0.5% for the doped samples (the same as for sample 3) but was increased to 0.75% for the undoped sample. This sample also showed the laterally grown region to be defective. Nevertheless, the distribution of these defects was clearer, and the observations showed that dislocations were generated in the doped layers. This is even more apparent in the more highly magnified region of the inset, where white arrows show some of these dislocations. These dislocations are revealed as white points, indicating that they lay into the growing plane. Their behaviour corresponded to that previously reported by Alegre et al. [15], where, first, the boron proximity effect generated dislocations into the growing plane, and, second, interaction with other dopants favoured their bending to thread across the epitaxial layer structure. Their Burgers vectors were $b=\frac{1}{2}\langle 011\rangle$ and $b=\frac{1}{6}\langle 211\rangle$, of the same vector family as that for sample 3.

## 4. Discussion

From this study, it seems evident that selective lateral growth can only be achieved using low methane concentrations. The use of CH_4_/H_2_ concentrations equal to or higher than 0.5% resulted in the generation of threading dislocations. Such dislocations could be $b=\frac{1}{2}\langle 011\rangle$ and/or $b=\frac{1}{6}\langle 211\rangle$. Alegre et al. [15] reported a critical boron level (CBL) in diamond samples depending on the CH_4_/H_2_ molar ratio and on the growth directions. They argued that substitutional boron atoms too close to each other (proximity effect) generate local stress on the diamond lattice. Such local stress results in the displacement of a carbon atom and the subsequent generation of a threading dislocation. This local stress can also block the dislocations, and this may explain why two different families of Burgers vectors were observed. During growth, $b=\frac{1}{2}\langle 011\rangle$ perfect dislocations are generated. It is energetically favourable for such dislocations to be dissociated into two $b=\frac{1}{6}\langle 211\rangle$ partial Shockley dislocations. When the boron concentration is too high, the stress generated by boron atoms blocks the dislocations and prevents their dissociation. This tentatively explains why, at high doping levels, only perfect dislocations with a $b=\frac{1}{2}\langle 011\rangle$ Burgers vector were observed.

**Figure 4:**
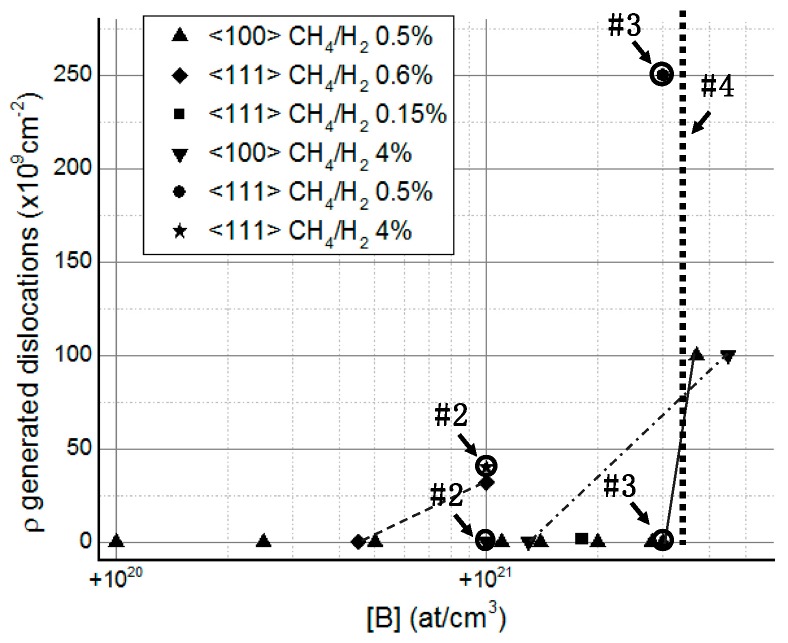
Density of dislocations as a function of the boron doping level of p^+^ layers obtained in [15], where circles correspond to samples grown along the <100> direction and stars correspond to samples grown along the <111> direction. Points highlighted by black circles correspond to the samples studied in regions grown along the <100> and <111> directions, respectively. Results from sample 4 are represented as a vertical dashed line, because it was not possible to determine the density of dislocations generated on each single orientation.

More evident in the micrographs (Figure 3c), this local stress also explains the behaviour observed for sample 4, for which dislocations were clearly generated in the doped layers. In this sample, the doping level was estimated at 2.3 × 10^21^ atm·cm^–3^. For similar growth conditions, the calculated CBLs were 6.5 × 10^20^ and 3.2 × 10^21^ atm·cm^–3^ for the <111> and <100> growth directions, respectively. Sample 4 was thus below the CBL for the <100> direction and above this value for the <111> orientation. A similar situation took place for samples 2 and 3, in good correspondence with the literature. Figure 4 shows the density of dislocations as a function of the boron doping level of p^+^ layers reported in the work of Alegre [15] and complemented by the present results. In the values extracted from the literature, each orientation, marked by stars or circles depending on whether they correspond to the <111> or <100> growth direction, respectively, was obtained from the study of a different sample. Here, the dislocation density generated along two different growth orientations was measured on the same sample, that is, under exactly the same growth conditions. Our results thus allow the appropriate growth parameters for good crystal quality in both planar and lateral growth to be limited somewhat more.

The present study shows that it is possible for growth along the <100> orientation with CH_4_/H_2_ of 0.5% without dislocations when boron concentration values are less than or equal to 3 × 10^21^ [B] atm·cm^–3^. However, as doping increased up to 4 × 10^21^ [B] atm·cm^–3^, the dislocation density increased quickly. Nevertheless, under the same growth conditions, samples grown along the <111> orientations were already shown to be full of defects at doping values of 3 × 10^21^ [B] atm·cm^–3^. Thus, for such growth orientations, the doping level should be reduced. Indeed, we note that, even at the low methane content of CH_4_/H_2_ of 0.15%, for growth along the <111> orientation, dislocations were observed. However, the dislocation density was much smaller even if the boron concentration was as high as ~10^21^ atm·cm^–3^.

## 5. Conclusions

This work confirmed the important role that the boron proximity effect has in the generation of dislocations in diamond growth. Selective diamond growth was shown to be possible at a low methane concentration. In fact, at 0.1% CH_4_/H_2_, diamond grew 12 times faster along the <111> direction than along the <100> direction, and without dislocations. Unfortunately, these growth rates are too slow for practical applications. Increasing the methane concentration results in faster growth that also affects the selectivity. However, for values of CH_4_/H_2_ of 0.5%, <111>-oriented growth was still twice as fast as that along the <100> direction. Nevertheless, selective growth was shown to also be highly restricted by the concentration of boron in the layer. Growth conditions should thus be chosen in terms of the methane concentration and boron doping levels in agreement with the selectivity required and the corresponding CBL.

## References

[1] Tolbert L.M., King T.K., Ozpineci B., Muralidharan G., Rizy D.T., Sabau A.S., Zhang H., Zhang W., Yu X., Huq H.F. (2005). Power Electronics for Distributed Energy Systems and Transmission and Distribution Applications: Assessing the Technical Needs for Utility Application.

[2] Yole Développement (2017). Power SiC: Materials, Devices, Modules, and Applications Report.

[3] Huang W., Chow T.P., Yang J., Butler J.E. (2004). High-voltage diamond Schottky rectifiers. Int. J. High Speed Electron. Syst..

[4] Spear K.E., Dismukes J.P. (1994). Synthetic Diamond: Emerging CVD Science and Technology Handbook.

[5] Traore A., Muret P., Fiori A., Eon D., Gheeraert E., Pernot J. (2014). Zr/oxidized diamond interface for high power Schottky diodes. Appl. Phys. Lett..

[6] Fang H., Katagiri M., Miyake H., Hiramatsu K., Oku H., Asamura H., Kawamura K. (2014). Crack-free GaN grown by using maskless epitaxial lateral overgrowth on Si substrate with thin SiC intermediate layer. Phys. Stat. Sol. A.

[7] Gupta A., Jacob C. (2005). Elective epitaxy and lateral overgrowth of 3C-SiC on Si—A review. Prog. Cryst. Growth Charact. Mater..

[8] Pinčík E., Kobayashi H., Brunner R., Takahashi M., Liu Y.L., Ortega L., Imamura K., Jergel M., Rusnák J. (2008). Passivation of defect states in Si-based and GaAs structures. Appl. Surf. Sci..

[9] Wang J., Youtsey C., McCarthy R., Reddy R., Allen N., Guido L., Xie J., Beam E., Fay P. (2017). Thin-film GaN Schottky diodes formed by epitaxial lift-off. Appl. Phys. Lett..

[10] Thomson S. (1998). MOS scaling: Transistor challenges for the 21st century. Intel Technol. J..

[11] Li F., Zhang J., Wang X., Zhang M., Wang H. (2017). Fabrication of Low Dislocation Density, Single-Crystalline Diamond via Two-Step Epitaxial Lateral Overgrowth. Crystals.

[12] Bauer T., Schreck M., Stritzker B. (2007). Epitaxial lateral overgrowth (ELO) of homoepitaxial diamond through an iridium mesh. Diam. Relat. Mater..

[13] Wang Y., Chang X., Liu Z., Liu Z., Fu J., Zhao D., Shao G., Wang J., Zhang S., Liang Y. (2018). Lateral overgrowth of diamond film on stripes patterned Ir/HPHT-diamond substrate. J. Cryst. Growth.

[14] Lloret F., Araujo D., Eon D., Villar M.P., Gonzalez-Leal J., Bustarret E. (2016). Influence of methane concentration on MPCVD overgrowth of 100-oriented etched diamond substrates. Phys. Stat. Solidi A.

[15] Alegre M.P., Araujo D., Fiori A., Pinero J.C., Lloret F., Villar M.P., Achatz P., Chicot G., Bustarret E., Jomard F. (2014). Critical boron-doping levels for generation of dislocations in synthetic diamond. Appl. Phys. Lett..

[16] Fiori A., Thi T.N.T., Chicot G., Jomard F., Omnes F., Gheeraert E., Bustarret E. (2012). In situ etching-back processes for a sharper top interface in boron delta-doped diamond structures. Diam. Relat. Mater..

[17] Lloret F., Araujo D., Alegre M.P., Gonzalez-Leal J.M., Villar M.P., Eon D., Bustarret E. (2015). TEM study of defects versus growth orientations in heavily boron-doped diamond. Phys. Status Solidi A.

